# Knowledge and use of therapeutic hypothermia in cardiac arrest victims among healthcare staff in Greece

**DOI:** 10.1186/cc13685

**Published:** 2014-03-17

**Authors:** P Manthou, T Katsoulas, A Korobeli, L Kiriakou, G Fildissis

**Affiliations:** 1 Department of Nursing University, Athens, Greece; 2 Gonk Agioi Anargiroi Hospital, Kifissia, Greece

## Introduction

Therapeutic hypothermia (TH) improves the neurologic outcome of patients who survive after cardiac arrest but suffer from severe secondary neurological damage [1]. In 2010, the use of TH after cardiac arrest was included in the ERC guidelines. The purpose of this study was to investigate the knowledge of the medical and nursing staff on the implementation of TH in patients after cardiac arrest [2].

## Methods

Data were collected by an anonymous questionnaire designed for the purpose of research, addressed to medical and nursing staff of Greek hospitals. The questionnaire consisted of questions about knowledge and behavior related to TH induction, target temperature and duration of cooling. Information about the potential barriers to implementation of TH was also collected.

## Results

We obtained 344 questionnaires from 16 hospitals. The population of the study was registered nurses (RN) (63.8%) and doctors (36.2%). The majority of health staff (81.5%) had never implemented TH. A total 45.8% of respondents stated that the main reasons for not using TH were the lack of information and training about the method, the lack of nursing staff, the lack of available cooling methods and the required time. The most common methods of application were cold packs and intravenous fluids. Only 30.2% of the doctors and 5.5% of the nurses (*P *< 0.001) actually had the knowledge to implement TH, and this was demonstrated by correct answers. Of the respondents who answered that they did know the method, only 23.9% answered correctly; about the target temperature, the maintenance and rewarming phase. A total 59.1% of doctors, despite having attended the Advanced Cardiac Life Support seminar, were not able to answer correctly the knowledge questions. Continuous education of health professionals and the existence of a protocol were proposed by 65% of participants as the best way of increasing knowledge and adherence with ERC guidelines about TH.

## Conclusion

Therapeutic hypothermia is rarely used in Greek hospitals. The level of knowledge is mainly related to the lack of education and the lack of information about new techniques. Programs for continuing education are necessary for the use of new therapeutic techniques in the field of health.
